# Examining Social Media User Types and the Impact on a Reproductive Health Web-Based Intervention for Young People in Francophone West Africa: Randomized Factorial Design Using Latent Class Analysis

**DOI:** 10.2196/83562

**Published:** 2026-07-23

**Authors:** Leona M Ofei, Nikolas Wianecki, Kyeongwon Kim, Catherine Crespi, Deffa Wane, Rabiatou Sangare, Alexandre Rideau, Mbathio Diaw, Philip M Massey

**Affiliations:** 1 Department of Community Health Sciences, Fielding School of Public Health, University of California, Los Angeles, 650 Charles E Young Drive S, 3rd Floor CHS Department, Los Angeles, CA, 90095, United States, 1 424-259-5954; 2 Department of Biostatistics, Fielding School of Public Health, University of California, Los Angeles, Los Angeles, CA, United States; 3 RAES: Réseau Africain de l'Education et de la Santé, Dakar, Senegal

**Keywords:** health communication, digital health, sexual and reproductive health, social media and health, web-based intervention

## Abstract

**Background:**

Web-based interventions present an opportunity for addressing reproductive health outcomes among young Francophone West Africans, but little is known about how social media use impacts such interventions.

**Objective:**

This study aimed to identify social media use patterns among young French-speaking West Africans and assess the impact on a reproductive health intervention delivered on Facebook.

**Methods:**

French-speaking social media users (aged 15‐24 years) were recruited from Senegal, Côte d’Ivoire, and Burkina Faso via social media advertisements. Using a factorial design for a multiarm parallel study, participants were randomly assigned via block randomization to 4 conditions: comparison (standard content), peer role models (personal narratives from similarly aged peers), online influencers (popular online figures), and a mixed peer role model and online influencer strategy. Participant blinding was implemented. Respondents completed pre- and post- self-administered online surveys between December 2023 and February 2024. Latent class analysis using maximum likelihood estimation was used to examine social media use patterns and related outcomes: contraceptive knowledge, provider awareness, and online service use.

**Results:**

In total, 353 participants were randomized to each group, with a final analytic sample of 262 participants. Two social media user types were identified from the completed study: selective users (daily use of WhatsApp and Facebook) and multiplatform users (daily use of WhatsApp, Facebook, Instagram, YouTube, and TikTok). Being 20-24 years old, compared with 15‐19 years, was associated with increased odds of multiplatform use (odds ratio [OR] 3.61, 95% CI 1.46‐9.36; *P*=.008). Furthermore, residing in Senegal was associated with a greater likelihood of multiplatform use, compared with Côte d’Ivoire (OR 0.27, 95% CI 0.08‐0.88; *P*=.03) and Burkina Faso (OR 0.23, 95% CI 0.07‐0.76; *P*=.02). Among selective users (daily users of WhatsApp and Facebook), the peer role model approach demonstrated a 1.84-unit increase in contraceptive knowledge (OR 1.84, 95% CI 0.27‐3.41; *P*=.02), a 1.81-unit increase in provider awareness (OR 1.81, 95% CI 1.15‐2.47; *P*<.001), and a 1.49-unit increase in service use (OR 1.49, 95% CI 0.06‐2.93; *P*=.04), compared with the comparison group. For multiplatform users, the peer role model approachwas associated with a decrease in provider awareness (OR 0.33, 95% CI −0.64 to −0.01; *P*=.04). Unlike the peer role model approach (n=70), generally, online influencer (n=63) and mixed approaches (n=57) did not yield significant outcomes, relative to the comparison group (n= 72). There were no adverse events reported.

**Conclusions:**

This study applies latent class analysis in a novel way for this population: examining how a comprehensive measure of social media use moderates digital interventions. This approach is underused in other social media studies in sub-Saharan Africa. Findings suggest that a one-size-fits-all approach in web-based interventions is not ideal, and online peer-to-peer communication is still an important strategy for certain social media users.

## Introduction

Sexual and reproductive health (SRH) is a public health priority in West Africa. In this region, among an estimated 33 million women of reproductive age who desire to avoid pregnancy, 56% do not have access to or do not use modern contraceptive methods [1,2]. These levels of unmet need are higher for adolescents and young women aged between 15 and 19 years (64%), making young adults a priority population [1,2]. These rates emphasize the need to advance SRH in this region, given that the average unmet need for modern contraceptives for young women is about 40% on average across all low- and middle-income countries compared with 23% on average in higher income countries [1,2]. In addition, public health organizations have called for increased attention to Francophone West Africa, given low contraceptive use trends and current levels of investment in the region [3,4]. For example, young women aged 15‐19 years in this Francophone region report average rates of unmet need for modern contraceptives that are similar to or higher than rates for young adults in other sub-Saharan African nations [1,2]. However, research related to SRH for young people in this age range has focused on English-speaking nations with limited focus in French-speaking West Africa [5]. Barriers to contraceptive use in this region have been tied to a lack of knowledge around contraceptives, limited awareness and access to service providers and family planning services, stigma and community norms around premarital sex, misinformation around the effects of contraceptives, pressure from male partners to have children, and financial limitations [6-9]. Therefore, SRH interventions are needed to address varying determinants that span across interpersonal, societal, and structural issues.

Digital health interventions present an opportunity to improve SRH outcomes in Francophone West Africa, given the steady growth of internet and social media use in sub-Saharan Africa, particularly among young adults [10]. In recent years, interventions in Francophone West Africa have leveraged the internet or social media, messaging platforms, chatbots, and online video content, and these strategies have been associated with improvements in SRH knowledge, awareness, attitudes, and self-efficacy [11-15]. This emphasizes the promise of digital interventions for addressing SRH literacy and behavior in the Francophone West African region.

The growth in internet and social media use and its ability for accelerated information dissemination opens avenues for exposure to both health-promoting information and misinformation, and many researchers continue to parse out these dual and contradictory outcomes [16-18]. For example, general internet and social media use has been identified as a potential space for online peer group discussion, as a source of increased access to SRH information, and as a channel to facilitate greater agency in contraceptive decision-making for women in sub-Saharan Africa [19,20]. At the same time, research has found a link between internet and social media use and risky sexual behavior in sub-Saharan Africa [21,22]. These mixed findings call for continued research to clarify the association between social media use and SRH behavior. However, limited studies in this region have considered a more comprehensive measure of social media use that is inclusive of a wide range of social media platforms. A more comprehensive approach could provide clarity on how social media use patterns are differentially related to SRH outcomes.

The Uses and Gratifications Theory offers a promising framework to understand how media use patterns influence health outcomes. This theory posits that media exposure is not a passive process, and users actively select the media they engage with based on certain needs and goals [23]. Scholars have found the framework to be useful when determining motivations behind social media use, ranging from information-seeking to entertainment to socialization [24,25]. Specifically, the type of content used, or the platform leveraged in an intervention, may have varied effects on various individuals with distinct social media use needs and motivations [26,27]. However, limited research has explored the efficacy of digital interventions in a media landscape where individuals are simultaneously exposed to a vast array of unverified health information from different social media platforms online [28]. This is especially critical as scholars point to media saturation as a threat to effective health communication, especially as individuals experience an overload of information and messaging fatigue [29,30].

In line with the Uses and Gratifications Theory, needs and motivations of social media use can be shaped by demographic contexts such as age and gender [31-33]. For example, research suggests that women use social media for maintaining relationships or for information and educational purposes, while men use these platforms for meeting new people [31,33]. Work in this field also suggests that social media use for younger people, compared with people who are older, may be driven by motivations to meet new people, maintain relationships, engage in entertainment, and access varied forms of information [31,32]. Examining these relationships is important as it could ultimately inform platform choice and messaging strategy targeted to different demographic groups with different digital media exposure needs and motivations. Further, identifying a comprehensive pattern of social media use that considers varying levels of engagement with different platforms will provide a more accurate reflection of daily social media engagement. Studies have used a latent class analysis (LCA) approach to identify unobserved groups that differ by their social media use patterns [34-37], although this is limited inthe West African context.

Therefore, this study will examine social media use patterns in a sample of Francophone West African young adults to address the following gaps: (1) the need for a more comprehensive assessment of social media use in this population that considers a wide range of social media platforms, (2) the value of additional studies to clarify inconsistent findings on the distinct social media use needs and motivations for various demographic groups, and (3) the lack of studies examining the efficacy of digital interventions within the context of social media use despite varied population needs and motivations and increasing media saturation. For this analysis, social media use patterns will be examined via an LCA approach to enable the identification of unobserved groups in our sample that differ by their social media use patterns [34-37]. Identifying a comprehensive pattern of social media use that considers varying levels of engagement with different platforms might provide a more accurate reflection of daily social media engagement in our sample, as opposed to assessing platforms individually.

The digital health web-based interventions to be considered in this analysis were implemented in a study to examine the influence of peer role model and online influencer approaches in addressing SRH health literacy and behavior among participants who were at the receiving end of the intervention. Findings in other parts of the world suggest that digital influencers have the potential to shape health literacy and behavior for better or worse [38-40]. However, more research is needed to explore the efficacy of online influencers for health promotion, particularly in the context of general social media use in French-speaking West Africa.

This was a stand-alone intervention targeted to adolescents and young adults in Burkina Faso, Senegal, and Côte d’Ivoire with the goal of testing its feasibility, efficacy, and potential for future health campaigns by the nongovernmental organization (NGO) Réseau Africain de l’Éducation pour la Santé (RAES), which translates to “African Network of Health Education” in English. The comparison exposure comprised standard content without external endorsements or personalization that reflected typical SRH material that the organization (RAES) shares online. The choice of this comparison exposure was to enable researchers to decipher whether newly tested approaches would be more efficacious or detrimental compared with standard content.

Overall, exploring the potential of digital interventions to address adolescent and youth SRH behavior within the context of social media exposure is important. It presents an opportunity to identify targeted strategies to address the low use of contraceptive methods among young adults in this region and expand access to SRH knowledge and resources. Research of this kind can inform interventions that could go a long way in curtailing the spread of sexually transmitted infections and in decreasing rates of unintended pregnancies, especially among adolescents and young adults in this region.

Thus, this study is guided by the following objectives: (1) to identify the underlying social media engagement patterns in a sample of French-speaking West African adolescent and young adult (aged 15‐24 years) social media users, (2) to examine the association between sociodemographic characteristics and social media use patterns in this sample, and (3) to assess whether social media use patterns influence the effect of a digital intervention for contraceptive knowledge, awareness, and practices, compared with a comparison group.

## Methods

### Open Science

This study was a pilot study for a local NGO to identify potentially effective strategies for recruitment, implementation, and evaluation of online health communication campaigns using social media in a resource-limited setting. The study was registered retroactively at Clinicaltrials.gov: NCT07636629.

### Research Study

This pilot study examined the influence of peer role model and online influencer approaches in addressing contraceptive knowledge and related behaviors, building off prior research [38-40]. The web-based intervention targeted adolescents and young adults in Burkina Faso, Senegal, and Côte d’Ivoire with the goal of testing its feasibility, efficacy, and potential for future health campaigns by the NGO, RAES. We pre-tested the proposed interventions with selected members of the public who fit the study criteria during the content development phase.

#### Sampling and Recruitment

From August to October 2023, we recruited a convenience sample of adolescents and young adults in West Africa using social media advertisements (primarily on Facebook). In total, 7013 participants expressed interest by completing the initial screening survey, but only 1412 of them met eligibility requirements for randomization (ie, those aged 15‐24 years; residing in Burkina Faso, Côte d’Ivoire, or Senegal; having internet access; having a verifiable Facebook account; having nonduplicate responses; and French speaker and reader). To identify and eliminate duplicate responses, we considered duplicate IP addresses, email addresses, and phone numbers. In addition, the team engaged in a thorough process of manually checking each account that passed the initial eligibility screener to identify indicators of a real and active account, such as personal profile details and signs of an active history. This is an online intervention, and there were no criteria for sites and for individuals delivering the interventions.

#### Study Design

The multiarm parallel study used a randomized factorial design via block randomization to allow for balanced representation across the main demographic variables (age group, gender, and country). The study statistician (CC) generated the random allocation sequence and assigned participants to the intervention groups. Only the study statistician (CC) had access to the random allocation sequence, which was maintained electronically in a spreadsheet. Specifically, participants were stratified into 18 strata based on these variables. Then, within each stratum, respondents were randomly assigned into the 4 intervention groups (comparison, peer role model, online influencer marketing, and mixed peer and online influencer approach) using permuted blocks of size 4. Overall, the study took on an exploratory approach to examine the impact of these proposed interventions, relative to a comparison group.

The study exposure group allocation ratio at randomization was 1:1:1:1 (n=353 participants in each group at randomization and approximately n=120‐125 participants in each exposure group at enrollment). These ratios, however, changed with attrition throughout the study, which explains the study group ratios for the baseline sample in this analysis, depicted in Table 1. The rest of the study team supported various phases of enrollment primarily led by DW and NW and did not have access to the random allocation sequence.

**Table 1.** Characteristics of the analytic sample of young adults in Francophone West Africa participating in a sexual and reproductive health digital intervention (N=262).

Characteristics	Participants
Age group (years), n (%)	
15‐19	65 (24.8)
20‐24	197 (75.2)
Gender, n (%)	
Man	124 (47.3)
Woman	138 (52.7)
Country, n (%)	
Senegal	126 (48.1)
Burkina Faso	63 (24.0)
Côte d’Ivoire	73 (27.9)
Level of education, n (%)	
Secondary or other	41 (15.7)
Technical school (école supérieure)	122 (46.6)
College/university or higher	99 (37.8)
Indicated daily use of platforms**^a^**, n (%)	
WhatsApp	235 (89.7)
Facebook	151 (57.6)
Instagram	116 (44.3)
YouTube	92 (35.1)
TikTok	61 (23.3)
Intervention, n (%)	
Comparison	72 (27.5)
Peer role model	70 (26.7)
Online influencer	63 (24.1)
Mixed peer role model + Influencer	57 (21.8)

a Percentages for daily use of social media platforms are out of the total (N=262). Denominator includes observations with missing data.

Participants were sent an invitation to the private Facebook group that corresponded to their assignment, and this invitation did not reveal whether they were in the intervention versus the comparison group. Attrition postrandomization was due to several factors. First, the potential for inconsistent access to the internet has been reported in other studies of digital health interventions in this region [41,42]. This likely meant that some participants may have faced challenges completing the pre- and/or postsurveys. These structural issues also meant an extended period of recruitment, verification, and enrollment, which yielded itself to a greater likelihood of participants dropping off during the study period.

Recruitment and verification stages were fully complete prior to the implementation of the study, and there were no changes to the methods once the study was implemented. However, we have considered additional analytical approaches such as the LCA.

#### Sample Size Determination

The sample size for this pilot study was selected based on the primary objective of comparing outcomes between groups using the SAS POWER procedure. We determined that a sample size of 200 participants per group would provide 80% power to detect a standardized mean difference between groups of 0.3, using an α value of .05 and after accounting for 10% attrition.

#### Data Collection

The randomly assigned participants were invited to join private Facebook groups (based on their intervention assignment) where the study content would be delivered. Participants were blinded to their interventions in that they did not know which exposure groups were the intervention groups versus the comparator group, as information sheets did not differ for the different exposure groups. However, participant blinding is challenging to implement in web-based trials [43], especially one of this kind whereby participants viewed content directly. The study (including enrollment, data collection, and intervention delivery) was between December 2023 and February 2024. Participants received unique survey links via provided contact information such as email and phone numbers and were prompted to complete preintervention (baseline) and postintervention (end line) online surveys. Ultimately, 492 participants were enrolled in the study, and 403 participants completed the pre- and/or postsurveys on self-reported measures during the study period, even with recruitment challenges expected in resource-limited settings [41,42]. Given the short nature of the exposure (2 weeks), we did not conduct any interim analysis or determine stopping guidelines.

#### Intervention Details

The following intervention details follow the TIDieR (Template for Intervention Description and Replication) checklist [44]. The intervention was dubbed as the C’est La Vie: Beta I Intervention. This intervention was implemented to examine whether digital social and behavioral change approaches increased the motivation and ability of young people to act on decisions for their SRH. It also aimed to examine whether these approaches increased SRH service use. Participants in Senegal, Côte d’Ivoire, and Burkina Faso completed a 2-week web-based intervention delivered in closed Facebook groups for each exposure type. The intervention was developed by the Francophone West African NGO, RAES. Intervention materials included culturally informed content related to SRH across the following intervention strategies: comparison, peer role model, online influencer, and mixed peer role model and online influencer approaches. The comparison group was exposed to content without external endorsements or personalization ie. typical SRH material that the organization (RAES) shares online. The choice of this comparison exposure was to enable researchers to determine whether newly tested approaches would be more effective versus detrimental compared with the standard content. For this group, they were exposed to clips and excerpt videos from the C’est La Vie web series with captions to elicit discussion or to promote or caution against certain behaviors. The peer role model group was exposed to content featuring young people from the target population who shared their personal experiences and stories related to SRH and presented advice or lessons learned to viewers in a video format. These young people were termed as “peer role models” as they were everyday young people that study participants could relate to even if they did not know them. The online influencer category involved exposure to SRH information shared by known or popular social media figures from the 3 countries who had a substantial online following. For the influencer approach, more socially oriented content was implemented, including social media live videos where influencers would speak in real time, as well as skits where they acted out scenarios while maintaining key messaging. There were 6 peer role models and 6 influencers (1 man and 1 woman from each of the 3 countries to allow for representation of the target population). Finally, the mixed peer role model and online influencer group was exposed to a blend of content from the peer role model and online influencer strategies. The video content described in this section ranged from as short as 20 seconds to over 2 minutes, with live videos generally being around 20 minutes. In addition, across the influencer, role model, and comparison exposures, 2 additional types of visual content were posted that included the same content, such as posts with questions to stimulate discussion or infographics that provided facts and information related to topics such as contraceptive use and unintended pregnancies. For this content that cut across, the content posters varied (ie, posted by role models, influencers, or the organization). The interventions involved ensuring consistency of key messaging across all 3 exposure groups and the comparison group. However, messengers, tone, and narrative framing differed based on the intervention approaches described in this section. Content consisted of text, images, and videos, and new content was posted almost every day over the 2-week period, which served as a way to reengage participants in the Facebook group during the study period. There were no offline face-to-face components of the study. However, 2 individuals were designated to support basic moderation on the Facebook pages (eg, monitoring activity, responding if needed, and ensuring respectful use of the platforms) based on a message guide. Currently, the intervention-specific materials are not accessible at a public location or website. However, the excerpts for the comparison exposure come from the C’est La Vie web series, which can currently be found on the C’est La Vie Official YouTube Page [45]. Apart from the social media live videos, created content was not modified once implementation began. In terms of fidelity or adherence, the team developed and followed a detailed content calendar that included the type of content and the poster for each group and for each date over the 2-week period to ensure that posts were delivered as planned.

### Ethical Considerations

All study procedures of this pilot study were approved by the University of California, Los Angeles Institutional Review Board (23‐000752). Prior to participation, participants were presented with a study information sheet online, which provided details on the study, including its focus on SRH. The sheet also emphasized that participants could choose not to take part in the study or could discontinue participation at any point in the study without repercussion. Participants were paid in the form of phone call credits amounting to about US $3‐$4. Study data were deidentified prior to analysis and were stored securely to ensure participant privacy and confidentiality. In addition, no identification of individual participants in any images in this manuscript and supplementary material is possible.

### Measures

#### Social Media Use

Participants were asked how often they had used the following social media platforms in the past 30 days: Facebook, Instagram, TikTok, WhatsApp, and YouTube. Respondents indicated responses on a 5-point scale including the options: Never, A few times a month, Once per week, A few times per week, and Daily. For this analysis, we were interested in exploring consistent and frequent exposure; thus, responses were categorized into two groups: daily use (1) compared with less frequent use (0). Social media use latent class groups were identified based on participants’ daily use (compared with less use) of the 5 social media platforms listed in this paragraph. This measure was included in the presurvey.

#### Intervention Group

Participants were categorized based on their intervention group: (1) Comparison, (2) Peer Role Model, (3) Online Influencer, and (4) Mixed Peer and Online Influencer Approach.

#### Knowledge of Contraceptive Methods

Respondents reported on their awareness of various contraceptive methods (including condoms, intrauterine devices, pill, implants, emergency contraception, etc)and participants responded Yes (1) or No (0) to whether they knew of these contraceptive methods . Responses to each method were summed based on 12 items, to create a total knowledge score ranging from 0 to 12. Higher scores reflected a greater awareness of contraceptive methods. These items were adapted from the women’s questionnaire for phase 8 of the Demographic and Health Surveys Program [46]. This measure was self-reported and was included in both the presurvey and the postsurvey.

#### Service Provider Awareness

Respondents indicated whether they were aware of 3 known SRH service providers in Francophone West Africa who were also highlighted in the study content. Specifically, respondents indicated whether they had heard of the following 3 organizations: Mama Network, Lydia Conseil, and Marie Stopes. A total score was summed ranging from 0 to 3 where zero represented no awareness of any of the providers, and 3 represented awareness of all the providers. Thus, higher total scores indicated greater service provider awareness. These providers were selected for the study with the guidance of project leads on the ground, given their awareness of the main SRH organizations in our countries of study. This measure was self-reported and was included in both the presurvey and the postsurvey.

#### Digital Service Use

Respondents indicated whether they had used SRH digital services from a list of 6 services. These included reaching out to organizations via their WhatsApp, using chatbots, visiting websites for information, counsel or products, and calling service providers. A total digital service use score was summed ranging from 0 to 6, whereby zero represented no digital services used, and 6 represented the use of all services listed. Higher total scores indicated greater digital service use. These services were identified for the survey with the guidance of project leads on the ground, given their understanding of SRH services in our study regions. This measure was self-reported and was included in both the presurvey and the postsurvey.

*Covariates* for this study included age group (15-19 years and 20-24 years), gender (woman and man), country (Burkina Faso, Côte d’Ivoire, and Senegal), and level of education (secondary or other, technical school/école supérieure, and college/university). These measures were self-reported and collected in the recruitment survey and the presurvey. Harms were not systematically assessed, and no participants reported any adverse effect to the study team.

### Data Analysis

This study examines the change in SRH outcomes from baseline to end line for various social media user types, using an LCA. In total, 492 participants were enrolled, and 403 completed the pre- and/or postsurveys. Social media use patterns were collected only at baseline so that participants would not conflate their typical social media use at end line with the additional Facebook exposure they gained during the intervention. Therefore, this analysis focused on changes in outcomes from baseline to end line only for participants who filled out the survey that included questions on social media use at baseline (n=267).

The LCA used Full Information Maximum Likelihood, which is a robust method to account for missing data under the missing at random assumption. This approach excludes only those observations that have missing data on all observed latent class indicators [36,47,48]. Thus, the final analytical sample for this analysis included participants who had complete data for the demographic variables that were considered in this study and data for at least 1 of the 5 indicators used to estimate social media use latent class groups (n=262).

Data cleaning and demographic statistics were completed using Stata (version 18; StataCorp LLC) [49], while LCA as well as subsequent analyses was conducted using MPLUS (version 8.11; Muthén & Muthén) [50]. To address the first objective, we conducted an LCA to identify unobserved groups based on participants’ daily use of the following social media platforms: Facebook, Instagram, TikTok, WhatsApp, and YouTube. We estimated models with 2-5 latent classes and identified the best fitting model based on the following indicators: AIC (Akaike Information Criterion), BIC (Bayesian Information Criterion), SABIC (Sample-Size Adjusted Bayesian Information Criterion), Vuong-Lo-Mendell-Rubin Likelihood Ratio Test (VLMRT), Lo-Mendell-Rubin Adjusted Likelihood Ratio Test (LMRT), and the Parametric Bootstrapped Likelihood Ratio Test (PBLRT). These are the most consistently recommended model fit criteria; entropy was also reported as is general practice [34-37]. Following standard practice, the optimal model fit was determined by the lowest values of AIC, BIC, and SABIC, and significant results for the likelihood ratio tests outlined previously (VLMRT, LMRT, and PBLRT). Statistically significant results for the likelihood ratio tests indicate that the current class solution is a better fit compared with the solution with 1 less class [34-37].

For the second objective, we examined whether sociodemographic characteristics predicted latent class group membership (ie, the social media use groups identified in the first objective). For this, we examined the bivariate odds of membership in one class versus the reference class for age, gender, country, and education. These odds were calculated using the 3-step estimation approach that involved identifying the latent class groups before examining their associations with predictors, adjusting for the relevant class estimation uncertainties [51]. We used the 3-step approach to allow for more consistent interpretations of class membership, given that we were examining different predictors. Odds ratios (ORs) and CIs are reported.

**Figure 1.**
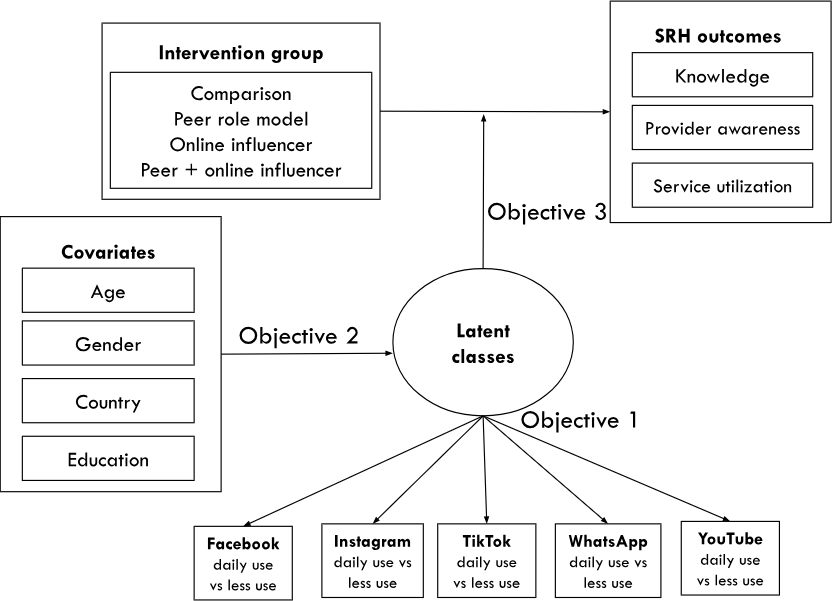
A conceptual model depicting the relationships examined among a sample of French-speaking West African young adult (aged 15‐24 years) social media users (N=262) including objective (1): the underlying social media use patterns identified via a latent class analysis, objective (2): the sociodemographic characteristics associated with identified social media use patterns, and objective (3): the moderating effect of social media use on the impact of a digital intervention on contraceptive knowledge, awareness, and service use. SRH: sexual and reproductive health.

To address the third objective, we examined whether the effect of the peer and online influencer interventions, compared with the standard comparison intervention, differed based on one’s social media use latent class group membership using maximum likelihood estimation and Monte Carlo integrations. This involved subgroup analysis to identify whether there was a significant difference in intervention effects, compared with the comparison group, within each latent class group. We also tested whether the outcome of the interventions in one latent class group was significantly different when compared with intervention outcomes in other latent class groups. The intervention outcomes for this analysis were maximum likelihood estimates reflecting changes in knowledge, service provider awareness, and service use from baseline to end line. Missing data in these analyses were accounted for using Full Information Maximum Likelihood, which is a robust method to account for missing data under the missing at random assumption [36,47,48]. The conceptual model (Figure 1) shows how the 3 objectives for this study guide our approach and analysis.

## Results

### Recruitment and Intervention Delivery

**Figure 2.**
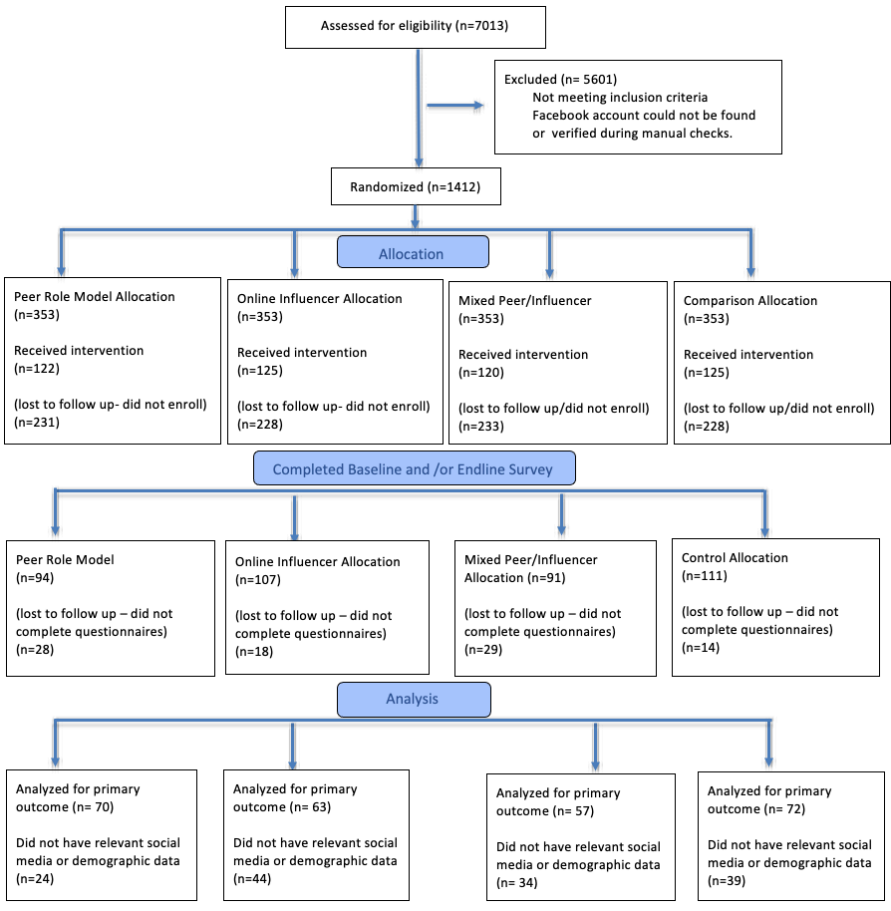
CONSORT (Consolidated Standards of Reporting Trials) 2025 flow diagram of the phases of a randomized factorial experiment (enrollment, allocation, survey completion, and data analysis) (adapted from Hopewell et al [52]).

Recruitment occurred from August to October 2023. After recruitment was completed, participants were randomized and then enrolled into the various intervention groups. Study implementation occurred between December 2023 and February 2024. This included enrollment, time for participants to fill out the baseline survey prior to the intervention, and the end line survey after the intervention. The intervention exposure itself was over a 2-week period in early to mid-February. The interventions were delivered as described in the “Methods” section. The intervention delivery followed a content schedule that the team followed so that each Facebook intervention group received respective content every day or every other day. The study did not end or stop unexpectedly and was implemented through completion. Participants did not receive concomitant care with this intervention. There were no harms measured or reported. In total, 353 participants were randomized to each intervention group and 492 participants enrolled to receive the intervention for the comparison (n=125), peer role model (n=122), online influencer (n=125), and the mixed approach (n=120). The final analytical sample (n=262) is based on relevant data availability as discussed in the “Data Analysis” section. The effects of the peer role model (n=70), online influencer (n=63), and the mixed peer and influencer intervention (n=57), compared with the comparison (n=72), were examined by social media user type for contraceptive knowledge, provider awareness, and service use. Figure 2 illustrates the sample sizes at each stage.

### Descriptive Results

This study focused on the sample of participants who had complete data for the demographic variables considered in this analysis and for at least 1 social media platform (N=262; Table 1). Overall, 75.2% (197/262) of the sample were aged between 20 and 24 years while 24.8% (65/262) were in the age range of 15-19 years. Gender distribution for women and men was 52.7% (138/262) and 47.3% (124/262), respectively. Overall, 48.1% (126/262) of this sample resided in Senegal, 24.1% (63/262) lived in Burkina Faso, and 27.9% (73/262) lived in Côte d’Ivoire. In terms of education, close to 15.7% (41/262) of this analytic sample had secondary school or other education, 46.6% (122/262) had attained tertiary technical school (école supérieur) education, and 37.8% (99/262) had attained college or university education or higher. Out of these 262 participants, 89.7% (235/262) of the sample indicated using WhatsApp on a daily basis while 57.6% (151/262) reported using Facebook daily. In addition, 44.3% (116/262) indicated daily Instagram use, 35.1% (92/262) reported daily YouTube use, and 23.3% (61/262) shared that they used TikTok daily. Additional descriptive results, stratified by intervention group, are included in Table S1 in Multimedia Appendix 1.

### Objective 1: Latent Class Group Estimation

#### Latent Class Group Estimation: Selecting the 2 Class Solution

To address the first objective, we examined model fit indices for the 2-5 class solutions derived from this analysis, described in Table 2. We selected the 2-class solution as the best fit for the sample in this analysis (AIC=1445.34; BIC=1484.60; LMRT=*P*<.001; VLMRT=*P*<.001; and BLRT=*P*<.001).

**Table 2.** Fit indices for the selection of a social media use latent class solution among the analytic sample of young adult social media users (aged 15‐24 years) in Francophone West Africa (N=262).

	2 Class	3 Class	4 Class	5 Class
AIC^a^	1445.34	1440.07	1443.47	1447.19
BIC^b^	1484.60	1500.74	1525.54	1550.67
SABIC^c^	1449.72	1446.84	1452.62	1458.73
Entropy	0.67	0.74	0.96	0.86
Vuong-Lo-Mendell-Rubin Likelihood Ratio Test for K-1 (H0) Versus K Classes^d^	*P*<.001	*P*=.03	*P*=.06	*P*=.02
Lo-Mendell-Rubin Adjusted LRT Test^d^	*P*<.001	*P*=.03	*P*=.07	*P*=.02
Parametric Bootstrapped Likelihood Ratio Test for K-1 (H0) Versus K Classes^d^	*P*<.001	*P*=.01	*P*=.25	*P*>.99
Class percentages	77.4, 22.6	24.4, 7.1, 68.5	6.9, 23.7, 57.6, 11.8	15.0, 1.2, 1.6, 7.1, 60.7

a AIC: Akaike Information Criterion.

b BIC: Bayesian Information Criterion.

c SABIC: Sample-Size Adjusted Bayesian Information Criterion.

d *P* values reported.

An assessment of the fit indices (Table 2*;* see “Data Analysis” section for criteria) narrowed down the potential choices to the 2 or 3 class solution. Then, the final selection of the 2-class solution came down to the BIC, AIC, *P* values for the likelihood ratio tests, and the interpretability of the 2-class solution in the wider geographic context [34-37].

For this analysis, AIC values were comparable between the 2-class (approximately 1445) and 3-class solutions (approximately 1440) while the 2-class solution showed a substantially lower BIC (approximately 1484) than the 3-class outcome (approximately 1500). In terms of the Vuong-Lo-Mendell-Rubin Likelihood Ratio, the Lo-Mendell-Rubin Adjusted LRT Test and PBLRT, both solutions showed evidence of good model fit at *P*<.05. However, when a more stringent *P* value benchmark was considered at *P*<.01, only the 2-class solution met this standard. Furthermore, LCA researchers recommend that each class should include at least 5%‐8% of the sample to ensure the stability and reliabilty of class solutions [36]. Thus, the 2-class solution was favored as the 3-class solution had 1 group that made up about 7% of the sample size.

We recognize that the 2-class model also showed the lowest entropy which could signal that there is less distinctness between the classes that resulted for this solution [36,53]. However, scholars indicate that entropy is not necessarily an appropriate measure of model fit [34,36,37,53]. Moreover, class solutions with higher entropy (4 and 5) did not perform better on other model fit criteria compared with the 2-class or 3-class solution. Furthermore, researchers suggest that entropy levels above 0.6 are considered reasonable [34,37,51]. Ultimately, scholars emphasize the importance of the interpretability of the class groups in the context of the real world [34,36,37]. As Bauer [34] puts it: “the goal is to yield a solution that balances model parsimony and fit and delivers substantively interpretable classes.” The 2-class solution is selected based on these criteria.

#### Latent Class Group Estimation: Description of Classes

**Figure 3.**
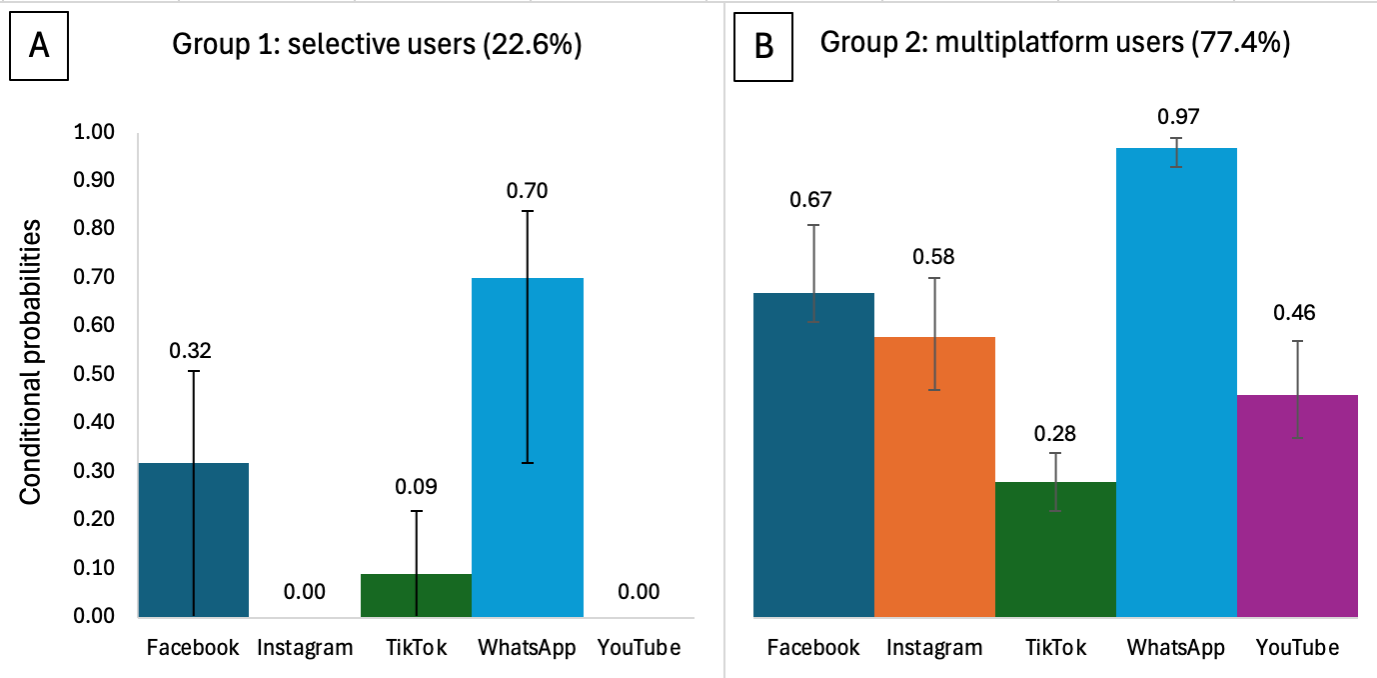
2-class solution showing the estimated daily social media use for the analytic sample of French-speaking West African young adult (aged 15‐24 years) social media users (N=262) via a latent class analysis. Error bars represent bootstrapped confidence intervals. (A) Likelihood of daily use of the various social media platforms for the selective social media user group. (B) Likelihood of daily use of the various social media platforms for the multiplatform social media user group. The bar graphs in Figure 3 compare the likelihood of daily use of social media between the 2 social media user types including daily use of Facebook (dark blue bar), Instagram (orange bar), TikTok (green bar), WhatsApp (light blue bar), and YouTube (purple bar).

The first latent class group (approximately 22.6% of sample) reflects those more likely to be selective social media users. Membership in this class is characterized by a high probability (70%) of daily WhatsApp use and a moderate probability (32%) of daily Facebook use. The probabilities of daily use of other platforms listed are negligible or close to 0. (9% for TikTok; approximately 0% for Instagram and YouTube). Participants most likely to belong to this class group will be referred to as selective users (Figures 3A and 3B).

The second latent class group (approximately 77.4% of sample) reflects those more likely to have daily exposure to multiple social media platforms. Membership in this class is characterized by a very high probability (97%) of daily WhatsApp use, a moderate to high probability of daily use of Facebook (67%) and Instagram (58%), a moderate probability of using YouTube daily (46%), and a moderate but lower probability of daily TikTok use (27%). Participants most likely to belong to this class group will be referred to as multiplatform users (Figure 3).

This 2-class solution fits the context of our region of study, given that WhatsApp and Facebook are the most popular platforms in sub-Saharan Africa, and WhatsApp is particularly amenable to regions with limited internet infrastructure [54-57]. Thus, it is possible that persons who are less active on social media in our sample (group 1: selective users) are likely to adopt these 2 platforms before any others. It also explains the high probability of daily use of WhatsApp, in particular, in both groups.

### Objective 2: Demographic Determinants of Latent Class Membership

For the second objective, results in Table 3 showed statistically significant bivariate associations of class membership with age group and with country. Specifically, being between 20 and 24 years, compared with being 15‐19 years, was associated with increased odds (OR 3.61, 95% CI 1.40‐9.36; *P*=.008) of being multiplatform users, compared with being selective users. In terms of country, residing in Burkina Faso (OR 0.23, 95% CI 0.07‐0.76; *P*=.02) and Côte d’Ivoire (OR 0.27, 95% CI 0.08‐0.88; *P*=.03), compared with residing in Senegal, was associated with 77% and 73% decreased odds, respectively, of being multiplatform users, compared with being selective users. There were no significant associations identified for gender and education level.

**Table 3.** Bivariate associations between sample demographics and social media use class membership for the analytic sample of young adult social media users (aged 15‐24 years) in Francophone West Africa (N=262).

Variable	Odds ratio	95% CI	*P* value
Odds of being a multiplatform user versus a selective user			
Age group (years)			
15‐19 (reference)	—^a^	—	—
20‐24	3.61	1.40-9.36	.008
Gender			
Man (reference)	—	—	—
Woman	2.14	0.86-5.34	.10
Country			
Senegal (reference)	—	—	—
Burkina Faso	0.23	0.07-0.76	.02
Côte d’Ivoire	0.27	0.08-0.88	.03
Education			
Secondary or other (reference)	—	—	—
Technical school / École supérieure	1.58	0.49-5.16	.45
College or university	1.87	0.54-6.51	.33

a Reference group category.

### Objective 3: The Moderating Effect of Latent Class Membership on the Impact of Intervention Approaches for Knowledge, Service Provider Awareness, and Service Use

Findings in Table 4 show that among selective users, exposure to the peer role model approach, compared with the comparison exposure, was significantly associated with an increase in knowledge by 1.84 units (95% CI 0.27-3.41; *P*=.02), an increase in service provider awareness by 1.81 units (95% CI 1.15-2.47; *P*<.001), and an increase in service use by 1.49 units (95% CI 0.06-2.93; *P*=.04).

**Table 4.** The moderation of social media use class membership on the impact of a digital sexual and reproductive health intervention for the analytic sample of young adult social media users (aged 15‐24 years) in Francophone West Africa (N=262): comparison (n=72), peer role model (n=70), online influencer (n=63), and mixed approach (n=57).

	Selective users		Multiplatform users		Interaction effect
Exposure group	MLE^a^ (95% CI)	*P* value^b^	MLE^a^ (95% CI)	*P* value^b^	*P* value^c^
Contraceptive knowledge change (0‐12)					
Comparison (reference)	—^d^	—	—	—	—
Peer role model	1.84 (0.27 to 3.41)	.02	0.38 (−0.33 to 1.10)	.29	.11
Online influencer	1.12 (−0.33 to 2.56)	.13	−0.09 (−1.08 to 0.89)	.85	.23
Peer + Online	1.66 (−0.01 to 3.32)	.05	0.54 (−0.23 to 1.30)	.17	.26
Service provider awareness change (0‐3)					
Comparison (reference)	—	—	—	—	—
Peer role model	1.81 (1.15 to 2.47)	<.001	−0.33 (−0.64 to −0.01)	.04	<.001
Online influencer	0.18 (−0.21 to 0.56)	.37	0.12 (−0.38 to 0.63)	.63	.88
Peer + Online	0.12 (−2.33 to 2.57)	.93	−0.14 (−0.78 to 0.49)	.66	.86
Digital service usage change (0‐6)					
Comparison (reference)	—	—	—	—	—
Peer role model	1.49 (0.06 to 2.93)	.04	−0.41 (−0.99 to 0.17)	.17	.02
Online influencer	0.46 (−0.46 to 1.38)	.33	−0.36 (−0.97 to 0.26)	.26	.18
Peer + Online	−0.46 (−2.04 to 1.12)	.57	−0.03 (−0.81 to 0.75)	.94	.66

a Maximum likelihood estimates (MLE) of continuous outcomes adjusting for age group, gender, country, education, and respective outcome baseline scores.

b Within-group *P* value, that is, the association between the intervention and the outcome within the subgroup.

c Testing whether there is a significant difference in the intervention effect between social media user subgroups.

d Reference group category.

Results for multiplatform users showed contrary results. Specifically, exposure to the peer role model approach, relative to the comparison exposure, was not significantly associated with a change in knowledge (95% CI −0.33 to 1.10; *P*=.29) and in service use (95% CI −0.99 to 0.17; *P*<.17) from baseline to end line. Moreover, this exposure, relative to the comparison exposure, was significantly associated with a decrease in service provider awareness by 0.33 units (95% CI −0.64 to −0.01; *P*=.04) from baseline to endline.

We also compared whether intervention outcomes were significantly different between the 2 groups (selective users vs multiplatform users). Results indicated that the effect of the peer role model approach was significantly different for selective users versus multiplatform users for service provider awareness (*P<*.001) and service use (*P*=.02). There was no significant difference in the intervention effect when comparing change in knowledge outcomes between both social media use groups.

In addition, the online influencer condition as well as the mixed peer role model and online influencer approach, when compared with the comparison exposure, was not associated with significant changes in outcomes for both types of users but with one exception. Specifically, results showed that the mixed peer role model and influencer approach, relative to the comparison, was associated with increased odds of knowledge gain, although these results were marginally significant (OR 1.66, CI −0.01 to 3.32; *P*=.051). All analyses adjusted for age, gender, country, education, and the baseline values for each respective outcome.

## Discussion

### Overview

This study sought to identify the underlying social media use patterns in a sample of French-speaking West African adolescent and young adult (aged 15‐24 years) social media users via an LCA. Results suggest that social media users in our sample can be categorized by 2 different types of daily social media engagement. These include selective users (daily use of WhatsApp and moderate Facebook use) and multiplatform users (daily use of all 5 platforms assessed, to varying degrees). We also examined the sociodemographic characteristics associated with social media use patterns and class membership. Our findings indicate that young adults aged 20‐24 years (compared with those aged 15‐19 years) are more likely to be multiplatform users, along with young adults residing in Senegal (compared with those in Côte d’Ivoire and Burkina Faso). Finally, we assessed whether social media use patterns influence the effect of a digital influencer intervention for SRH knowledge, awareness, and practices. Study findings show that the peer role model approach was associated with positive gains in knowledge, service provider awareness, and service use for selective users only. However, among multiplatform users, this exposure was not associated with significant changes for knowledge and service use. Furthermore, it was related to decreased gains for service provider awareness. Online influencer and mixed approaches did not show any effect for both groups in this sample for the most part although the mixed condition yielded some gains in knowledge for selective users. The implications of these findings are highlighted in this Discussion section.

### Daily Social Media Use 2-Class Solution: Selective vs Multiplatform Users

Selective users are characterized by daily use of WhatsApp and moderate Facebook use primarily while multiplatform users reflect daily use of all platforms (Facebook, Instagram, TikTok, WhatsApp, and YouTube) to varying degrees. Drawing from the Uses and Gratifications theory [23], the 2-class solution could be a representation of 2 groups with different needs and motivations for social media use. Research suggests that instant messaging users leverage these platforms for maintaining relationships while Facebook users leverage this platform to stay updated on the activities of people who are generally in their network. Both involve some level of motivation to stay connected to peers and other networks [25]. Additional research reinforces WhatsApp as a desired platform for seeking to build or maintain close relationships but suggests that Facebook seems to be geared more toward information gathering about one’s social connections [58]. Moreover, an exploration of multiple social media platforms found that Facebook (and LinkedIn) were the only platforms where young adults were influenced by peers to join, highlighting that there are peer-related motivations for using Facebook as well [59]. Furthermore, in a study that examined multiple social media platforms, researchers found that Instagram and TikTok were more strongly associated with motivations such as entertainment, escapism, connecting with likeminded people, and content creation opportunities [60].

These findings suggest that instant messaging platforms such as WhatsApp may meet the needs of people seeking to maintain close relationships. While Facebook also leans toward social connections in one’s network, these networks may be more expansive beyond close peers [25,58,59]. Thus, it is possible that the latent class group consisting of selective users in our sample uses social media to meet needs related to maintaining close relationships. This may be why selective users in this analysis have a high probability of daily WhatsApp use and a moderate probability of Facebook use, with negligible probabilities for other platforms. On the other hand, based on findings from Karapanos et al [58], we can infer that multiplatform users may use several platforms as their needs extend beyond social connection to motives such as entertainment and escapism.

These findings have implications for digital health interventions particularly as research suggests that the media use needs and motivations of individuals may interplay with the impact of health messages, depending on the mode of communication and the media channels used [26,27]. Thus, paying attention to distinct social media patterns, and the needs and motivations they address, may ultimately facilitate better tailored health messaging interventions.

### Demographic Determinants of Class Membership: Age and Country

Demographic differences (age and country) were associated with class membership in bivariate analysis. Those who were aged 20‐24 years, compared with participants who were aged 15‐19 years, were more likely to be multiplatform users, compared with being selective users. These findings align with prior work suggesting that different age groups have distinct social media use needs and motivations [31,32]. For our sample of young adults, one distinction could be that these groups are in different phases of emerging adulthood. Those in the age range of 15‐19 years tend to be those who may still have heavy parental involvement and whose main social connections are in more controlled settings such as high school. However, for those who are in the age range of 20‐24 years, they may be at a point in adulthood where their social networks are expanding, as they become more responsible for themselves and make job and career choices [61-64]. Thus, for the older group, their needs and motivations may become more expansive beyond close networks, which would amend itself to seeking multiple platforms to meet these growing and expanding needs.

Moreover, in this region, internet use is shaped by access to monetary resources, and WhatsApp is particularly popular, given its amenability to areas with limited internet infrastructure, compared with other platforms [57]. Thus, adolescents (aged 15-19 years) in limited resource settings, who may have less financial autonomy for frequent use of other platforms, may lean more toward using WhatsApp primarily, compared with young adults (aged 20-24 years) who may have more financial autonomy to explore other platforms.

Findings also indicated that residing in Burkina Faso and Côte d’Ivoire, compared with residing in Senegal, was significantly associated with decreased odds of being a multiplatform user, compared with being a selective user. Data show that as of 2023, Senegal had the highest rates of internet use, compared with Côte d’Ivoire and Burkina Faso (17%) [65]. Thus, these results likely reflect differences in internet infrastructure and access, which suggests that a greater population of people in Côte d’Ivoire and Burkina Faso, compared with Senegal, could be more limited to primarily WhatsApp use.

### Moderation Analysis: Peer Role Model Approaches Are Effective for Selective Users but Not for Multiplatform Users

Findings showed that exposure to peer role model approaches was associated with improved contraceptive knowledge, service awareness, and service use for selective users but not for multiplatform users. In line with the Uses and Gratifications theory [23], prior research suggests that preferences for building close relationships may motivate WhatsApp use [25,58]. Thus, selective users may respond more positively to the intervention that includes people they can more easily relate to and connect with on some level (even if they do not know them), particularly as these role models shared their personal stories and experiences.

The difference in the effects of the peer role model intervention for both users could also be related to how different users process the information they are exposed to. The Elaboration Likelihood Model of Persuasion posits that there are 2 paths that shape the persuasion process. The central route includes a more thorough and critical contemplation of messages, which allows for longer-lasting persuasion effects [66]. However, the peripheral route is less likely to elicit long-lasting effects as it involves less critical processing, lower cognitive requirements, and persuasion tends to be shaped by cues (eg, emotions leveraged or number of arguments presented) rather than a thoughtful contemplation of information [66]. This could be relevant to social media use patterns. Studies show that media and digital multitasking is associated with less focus and poorer learning and memory outcomes [67,68]. Perhaps, the distraction of media multitasking could make it harder for multiplatform users to fully process and understand the information they are exposed to, which could make certain health-messaging interventions less impactful for this group.

Thus, additional research should continue to explore strategies that can facilitate message processing for social media multiplatform users who may be less likely to engage in the central route of persuasion. For example, message repetition can allow for additional information processing but with threshold effects [66]. Overall, these findings emphasize that different messaging strategies should be used to target different types of social media users.

Our study did not detect significant changes in outcomes based on the influencer intervention for either type of user. This is contrary to findings that suggest that influencers shape health behavior positively or negatively [38-40]. Prior research has highlighted the importance of building audience trust for effective influencer and celebrity marketing approaches [69-71]. In addition, audiences consider the presence of paid marketing and endorsements in their assessment of influencer credibility [72,73]. Therefore, it is possible that participants did not trust some or all the influencers in this intervention for different reasons, which may have limited the potential of the intervention.

Furthermore, research suggests that people may form parasocial attachments to influencers they follow, which heightens their influence [74]. For this study, however, this was likely not the case since participants may not have felt connected to the group of influencers selected for the study. Perhaps an intervention that intentionally included influencers that participants considered as favorites would have yielded more promising results. Therefore, future research examining the potential of influencer marketing for public health should consider how to gauge participant perceptions of trust and emotional connection to selected influencers before proceeding with interventions.

Generally, the mixed peer role model and influencer approach did not yield significant results for this sample of social media users, although we noted marginally significant improvements in knowledge for selective users only. There are mixed findings on the efficacy of interventions with multiple components [75]. Compared with singular interventions, multifaceted approaches may involve multiple attention demands and cognitive overload, which limit their impact [76]. Thus, it is possible that exposure to both the peer and influencer interventions heightened cognitive burden, which diminished their potential benefits. This may be why the gains from the mixed approach were only marginally significant for knowledge among selective users only and not for any other outcomes and multiplatformusers. It may also be that the distrust of online influencers interfered with the perception of peer role models, given that participants in this group interacted with both strategies at the same time.

### Limitations

This study encountered some limitations. First, a majority of our sample resided in urban areas. Thus, this sample may not be entirely representative of the population of young adult social media users in French-speaking West Africa. Unfortunately, rural-urban internet service gaps remain in sub-Saharan Africa [77]; hence, persons who currently use social media platforms are more likely to be urban dwellers. Thus, these findings give us preliminary insight into how social media behavior can shape the impact of digital interventions among young adult social media users, which can be useful for future research as internet and social media use expand into rural regions. Furthermore, participant blinding is challenging to implement in web-based trials, considering that participants are aware of what they are viewing or interacting with [43]. While participants did not have specific knowledge of which exposures were the treatment versus the comparator, there may also have been social desirability present during survey completion, given their awareness of exposure to reproductive health content. It may also be possible that there are important social media platforms that were not considered in this analysis, as this study was a focused experiment on a specific target population in a more controlled research environment. This may limit the generalizability of findings to the general population of social media users.

Finally, our study was not without its recruitment and retention challenges, given the nature of research in resource-limited settings [41,42]. Therefore, our sample size was 267 at baseline (when social media use data were collected) and 262 for this analysis. Smaller sample sizes present the potential for less precise estimates [78], as is evident by some of the confidence intervals reported in these analyses, particularly for the demographic associations. Moreover, the confidence intervals characterizing the association between demographic characteristics and latent class membership are particularly wide because they reflect uncertainty in both latent class membership and the strength of the association. Thus, we emphasize the need for robust logistical and funding support for additional studies of this kind in resource-limited settings. This will be increasingly pertinent as internet and social media use expand among young adults in low- and middle-income regions [10]. Given our limited sample size, we present these results, interpret them with caution, present them as preliminary findings for future studies, and call for future research with more expansive and representative samples.

Our study presents some notable strengths. First, pre- and postsurvey data collection allowed for a clear temporal order in our analysis of change in outcomes. In addition, using the LCA approach provided a more comprehensive picture of social media use in the sample as opposed to examining independent platforms separately. Finally, working with partners on the ground through survey and intervention development, as well as data collection, allowed for our study to be suitable and relevant for our population of interest.

### Conclusions

Overall, this study offers insight into how general social media behavior and purposeful digital interventions interact to determine SRH outcomes in French-speaking West Africa. It moves the literature forward in an understudied yet important area of research in Francophone West Africa. Furthermore, it applies a novel approach to examining how a comprehensive measure of social media use moderates digital health interventions by using an LCA approach, which is underutilized for research in this population. Results show that a one-size-fits-all approach in web-based interventions is not ideal, and online peer-to-peer communication is still an important strategy for certain social media users but not for others. Findings are important for identifying tailored and impactful interventions that consider both demographic contexts and media exposure patterns. The findings also suggest that multiplatform social media use could be detrimental for the impact of health-messaging interventions. This has implications for future public health work as social media platforms continue to maintain popularity and proliferate. It aligns with work that indicates that increasing media saturation may pose a threat to effective health communication as people experience information fatigue and messaging overload [29,30]. This should incentivize health communication researchers to explore what strategies will cut through social media noise, especially for individuals who use multiple social media platforms on a frequent basis.

Given that these results serve as preliminary findings in an understudied research area in French-speaking West Africa, future studies should explore this area of research with larger and more representative samples to ensure consistency of results. Studies of this kind should also consider repeated measurement of outcomes over a longer period after the intervention to examine whether these strategies result in sustained health literacy and behavior changes.

## Supplementary material

10.2196/83562 Multimedia Appendix 1 Characteristics of this analytic sample of young adults in Francophone West Africa participating in a sexual and reproductive health web-based intervention by exposure group.

10.2196/83562 Checklist 1 CONSORT-EHEALTH checklist (V 1.6.1). From Eysenbach G; CONSORT-EHEALTH Group [79].

## Abbreviations:

AIC: Akaike Information Criterion
BIC: Bayesian Information Criterion
LCA: latent class analysis
LMRT: Lo-Mendell-Rubin Adjusted Likelihood Ratio Test
NGO: nongovernmental organization
OR: odds ratio
PBLRT: Parametric Bootstrapped Likelihood Ratio Test
RAES: Réseau Africain de l’Éducation pour la Santé
SABIC: Sample-Size Adjusted Bayesian Information Criterion
SRH: sexual and reproductive health
TIDieR: Template for Intervention Description and Replication
VLMRT: Vuong-Lo-Mendell-Rubin Likelihood Ratio Test
