# Supplementary material for: Examining Social Media User Types and the Impact on a Reproductive Health Web-Based Intervention for Young People in Francophone West Africa: Randomized Factorial Design Using Latent Class Analysis
Source: JMIR Form Res. 2026 Jul 23;10:e83562. doi: 10.2196/83562 (PMC13397007; doi:10.2196/83562)
Supplement: Multimedia Appendix 1 [file formative-v10-e83562-s001.pdf]

Multimedia Appendix 1

Table S1. Characteristics of the analytic sample of young adults in Francophone West Africa participating in a sexual and reproductive health web-based intervention by exposure group.

	Full Sample (N=262)	Control (N=72)	Peer Role Model (N= 70)	Online Influencer (N=63)	Mixed Intervention (N=57)
**Age Group**					
15-19	65 (24.8)	23 (31.9)	16 (22.9)	13 (20.6)	13 (22.8)
20-24	197 (75.2)	49 (68.1)	54 (77.1)	50 (79.4)	44 (77.2)
**Gender**					
Man	124 (47.3)	44 (61.1)	25 (35.7)	29 (46.0)	26 (47.3)
Woman	138 (52.7)	28 (38.9)	45 (64.3)	34 (54.0)	31 (54.4)
**Country**					
Senegal	126 (48.1)	38 (52.78)	33 (47.1)	29 (46.0)	26 (45.6)
Burkina Faso	63 (24.0)	13 (18.1)	17 (24.3)	18 (28.6)	15 (26.3)
Cote D'Ivoire	73 (27.9)	21 (29.2)	20 (28.6)	16 (25.4)	16 (28.1)
**Level of Education**					
Secondary or Other	41 (15.7)	14 (19.4)	9 (12.9)	7 (11.1)	11 (19.3)
Technical School (Ecole Supérieur)	122 (46.6)	29 (40.3)	35 (50.0)	31 (49.2)	27 (47.3)
College/University or Higher	99 (37.8)	29 (40.3)	26 (37.1)	25 (39.7)	19 (33.3)
**Indicated Daily Use of Platforms**[a]					
WhatsApp	235 (89.7)	61 (87.1)	67 (95.7)	56 (88.9)	51 (92.73)
FB	151 (57.6)	37 (54.4)	40 (58.0)	35 (55.6)	39 (72.2)
Instagram	116 (44.3)	32 (44.4)	31 (44.9)	26 (41.3)	27 (48.2)
YouTube	92 (35.1)	29 (40.9)	20 (29.9)	24 (38.1)	19 (35.2)
TikTok	61 (23.3)	12 (17.1)	21 (30.4)	15 (23.8)	13 (23.6)
